# Effectiveness of Sound Field Corrections for High-Frequency Pressure Comparison Calibration of MEMS Microphones

**DOI:** 10.3390/s25051312

**Published:** 2025-02-21

**Authors:** Fabio Saba, María Campo-Valera, Davide Paesante, Giovanni Durando, Mario Corallo, Diego Pugliese

**Affiliations:** 1 Istituto Nazionale di Ricerca Metrologica (INRiM), 10135 Torino, Italy; davide.paesante@gmail.com (D.P.); g.durando@inrim.it (G.D.); m.corallo@inrim.it (M.C.); d.pugliese@inrim.it (D.P.); 2 Telecommunication Research Institute (TELMA), Universidad de Málaga, 29010 Málaga, Spain

**Keywords:** MEMS microphone, pressure comparison calibration, acoustic field, sound field corrections, FEM, acoustic metrology, active coupler, insert voltage technique

## Abstract

The calibration of Micro-Electro-Mechanical System (MEMS) microphones remains a critical challenge due to their miniaturized geometry and sensitivity to non-uniform acoustic fields. This study presents an advanced calibration methodology that integrates Finite Element Method (FEM) simulations with experimental corrections to improve the accuracy of pressure comparison calibrations using active couplers. A key innovation is the incorporation of asymmetric acoustic field analysis, which systematically quantifies and corrects discrepancies arising from cavity geometry, sensor positioning, and resonance effects peculiar of MEMS microphones. The proposed approach significantly reduces measurement uncertainties, especially in the high-frequency range above 5 kHz, where standard calibration techniques face challenges in taking into account localized pressure variations. Furthermore, the implementation of a measurement set-up, which includes the insert voltage technique, allows for an accurate assessment of the preamplifier gain and minimizes systematic errors. Experimental validation shows that the refined calibration methodology produces highly reliable correction values, ensuring a robust performance over a wide frequency range (20 Hz–20 kHz). These advances establish a rigorous framework for standardizing the calibration of MEMS microphones, strengthening their applicability in acoustic monitoring, sound source localization, and environmental sensing.

## 1. Introduction

Micro-Electro-Mechanical System (MEMS) microphones have become essential components in a wide range of acoustic applications due to their compact size, low power consumption, and low-cost fabrication [1,2,3,4]. They are widely used in consumer electronics [5,6,7,8] and play a critical role in low-cost wireless sensor networks, enabling detailed soundscape analysis in urban and natural environments [9,10,11]. In addition, their compactness and integration capabilities make them invaluable for sound source localization [12,13,14,15] in critical applications, such as seismic event detection, traffic monitoring, and security systems [16,17,18,19].

Traditional pressure comparison calibration methods, such as those described in IEC 61094-5 [20,21], rely on reference microphones that are traceable to the International System of Units. However, these methods struggle to fully compensate for geometric differences between MEMS and reference microphones, resulting in systematic errors, especially at high frequencies. In addition, traditional approaches fail to account for the complex acoustic interactions within pressure couplers, affecting measurement repeatability and traceability.

To ensure the accurate calibration of MEMS microphones, the comparison with a reference microphone, whose sensitivity is determined through the primary calibration by the pressure reciprocity method, is essential. Active coupler-based calibration allows for simultaneous acoustic excitation of the MEMS and reference microphones, ensuring frequency responses to be determined over a wide range (20 Hz to 20 kHz). However, geometric discrepancies require precise correction methods to mitigate local pressure variations.

A major limitation of previous studies is the inadequate modeling of asymmetric pressure distributions in calibration set-ups. Although Finite Element Method (FEM) simulations have been applied [22], most implementations assume ideal axisymmetric conditions that do not reflect real-world measurement environments. This oversimplification neglects possible geometric asymmetries and off-axis microphone positioning effects, which might be important, especially at frequencies above 5 kHz, limiting the accuracy of correction factors. Real acoustic pressure distributions exhibit complex non-uniformities due to factors such as asymmetric sound radiation patterns and cavity geometry [23].

This paper presents a novel calibration methodology that integrates the FEM solution of the acoustic field under ideal axisymmetric conditions with an experimental framework to quantify and correct the asymmetric sound field effects in the active coupler; these effects typically occur in the comparison calibration of MEMS microphones against Laboratory Standard (LS) and Working Standard (WS) microphones. Unlike previous indirect estimation techniques, this approach uses high-resolution pressure mapping and experimental corrections to improve the calibration accuracy, bridging the gap between ideal and real conditions.

In addition, this methodology includes an innovative implementation of the insert voltage technique that improves gain estimation in MEMS microphone preamplifiers. This reduces systematic errors and ensures more consistent calibration results across different conditions. These advances set a new standard for MEMS microphone calibration, especially in the high-frequency range above 5 kHz.

Experimental results show that this refined technique significantly reduces uncertainty across the frequency spectrum, a significant improvement over conventional methods. Moreover, the results of this study highlight the potential of this approach to serve as a benchmark for future standardization efforts in MEMS microphone calibration, thereby improving reliability in scientific and industrial applications.

The integration of numerical simulations, experimental corrections, and innovative measurement set-ups improves the reliability of MEMS microphones and broadens their applicability in various contexts. This comprehensive approach solidifies MEMS microphones as a cornerstone technology in modern acoustics.

This paper is organized as follows: Section 2 explains the pressure comparison calibration and the evaluation of sound field corrections through both analytical and numerical modeling of the acoustic field. Section 3 describes the implementation of the pressure comparison method for the calibration of MEMS microphones by the active coupler. Specifically, the experimental method to compensate for the sound field asymmetries is detailed. In Section 4, the experimental results, concerning the evaluation of the sound field corrections for the pressure comparison calibration by the active coupler, are shown. Finally, Section 5 draws the main conclusions of this study.

## 2. Secondary Pressure Calibration of Miniaturized Microphones

The comparison method is an effective technique for calibrating non-conventional microphones, characterized by non-standard sizes and layouts, and plays an important role in providing metrological traceability for this type of acoustic transducer. It is a secondary calibration method, which means that traceability is ensured by a reference microphone whose sensitivity has been obtained by primary calibration methods, such as the reciprocity technique. In the case of miniaturized sensors, such as MEMS microphones, comparative calibration under pressure-field conditions is particularly valuable because it allows the frequency response to be characterized under experimental conditions that closely mimic the operating environment.

Issues related to the pressure comparison calibration of microphones of smaller size and different electroacoustic characteristics compared to the reference microphone have been addressed by several studies [24,25]. Moreover, guidelines for the calibration of ${¼}^{\prime \prime }$ WS microphones (WS3 type) against ${½}^{\prime \prime }$ LS microphones (LS2 type) are described by the international standard IEC 61094-5 [20]. It is crucial to note that in order to provide direct metrological traceability to primary standards of acoustic pressure, smaller reference microphones used for pressure comparison calibration—which can also be calibrated by the primary pressure-reciprocity method—must be of the ${½}^{\prime \prime }$ LS2 type. Therefore, a well-defined step-down procedure for the pressure comparison calibration of smaller microphones is necessary.

To this aim, for the calibration of conventional ${¼}^{\prime \prime }$ diaphragm diameter WS condenser microphones, analytical models are proposed to evaluate sensitivity corrections, compensating for the difference in acoustic pressure to which the reference (${\mathrm{Mic}}_{\mathrm{ref}}$) and test (${\mathrm{Mic}}_{\mathrm{test}}$) microphones are exposed [24,25]. This difference results from the non-uniform distribution of acoustic pressure within the narrow gap between the diaphragms of the facing microphones when the simultaneous excitation method is used. The discrepancy becomes more pronounced as the size differences and sound wave frequencies increase.

### 2.1. Analytical Solution

**Figure 1:**
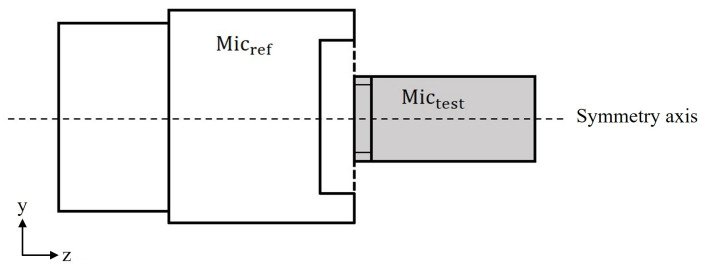
Coupling configuration of reference microphone (${\mathrm{Mic}}_{\mathrm{ref}}$) and test microphone (${\mathrm{Mic}}_{\mathrm{test}}$), where sound field corrections can be modeled analytically.

In a pressure comparison calibration using the simultaneous excitation method, the ${\mathrm{Mic}}_{\mathrm{ref}}$ and ${\mathrm{Mic}}_{\mathrm{test}}$ are placed face-to-face with their diaphragms coaxial with each other and with the sound source, separated by a distance much smaller than the acoustic wavelength, as shown in Figure 1. This configuration ensures that pressure field conditions are established over the frequency range of interest. These conditions can be achieved in a free-field configuration or, alternatively, by creating a uniform acoustic pressure distribution around the circumferential boundary of the gap between the microphones. This is typically achieved using active acoustic couplers with annular sources or in reverberation chambers.

In these simple implementations, if the ${\mathrm{Mic}}_{\mathrm{ref}}$ and ${\mathrm{Mic}}_{\mathrm{test}}$ are of the same type and dimensions, the sensitivity is determined by the ratio of open-circuit voltages produced by both microphones relative to the known sensitivity of the ${\mathrm{Mic}}_{\mathrm{ref}}$. In this case, since the diaphragms have the same diameter, both microphones are exposed to the same sound pressure, regardless of any acoustic field irregularities. Therefore, no additional corrections are necessary except for compensating for the differences in the voltage gains of the measurement chains and the environmental coefficients of the microphones. However, if the ${\mathrm{Mic}}_{\mathrm{test}}$ with a smaller sensing area than the ${\mathrm{Mic}}_{\mathrm{ref}}$ is calibrated by pressure comparison, corrections for acoustic field non-uniformities must be applied.

Sound field corrections (referred to as δ) are applied to the microphone sensitivity level and are defined as follows [25]:

(1) $$\delta =20\log_{10}\left(\frac{{\bar{p}}_{ref}}{{\bar{p}}_{test}}\right)$$

where ${\bar{p}}_{ref}$ and ${\bar{p}}_{test}$ represent the moduli of the acoustic pressure, spatially averaged over the detection areas of ${\mathrm{Mic}}_{\mathrm{ref}}$ and ${\mathrm{Mic}}_{\mathrm{test}}$, respectively, and weighed according to the normalized spatial distribution of the microphones’ sensitivities (where known).

The measurement model for determining the pressure sensitivity level $S_{test}$ in dB re 1 V/Pa of a test microphone using the comparison method, with the application of sound field corrections, can be expressed in the following form [22]:

(2) $$S_{test}=S_{ref}+L_{V}+\Delta G+\Delta E+\delta$$

$S_{ref}$ is the known sensitivity level of the ${\mathrm{Mic}}_{\mathrm{ref}}$; $L_{V}$ is the ratio, in dB, of the output voltages of the ${\mathrm{Mic}}_{\mathrm{test}}$ and ${\mathrm{Mic}}_{\mathrm{ref}}$ measurement chains; ΔG is the difference in voltage gains between the two measurement chains; and ΔE is the difference in environmental corrections between the two microphones, necessary to evaluate $S_{test}$ under the same ambient conditions (static pressure, temperature, and relative humidity) as $S_{ref}$.

The analytical approach is the most appropriate method for evaluating sound field corrections, since their experimental evaluation is impractical and infeasible. This would require non-intrusive sampling of the acoustic field by miniaturized sensors or non-invasive techniques that do not perturb the field itself. An analytical solution of the acoustic field was provided in [24,25] for a specific microphone installation configuration, which showed good repeatability of measurement results. It also allowed experimental validation using reference and test microphones whose pressure sensitivities were both known from the primary reciprocity calibration [24].

The analytical solution of the acoustic field allows the evaluation of mean acoustic pressures averaged over the sensing surfaces of the reference and test microphones, and the calculation of sound field corrections at different frequencies using Equation (1).

The analytical modeling of the acoustic field for this pressure comparison configuration is based on the assumption of an axisymmetric domain, where the acoustic pressure distribution is determined within the cylindrical space between the microphones, considering the annular open surface around the smaller microphone as the sound source boundary. This has been applied to evaluate sound field corrections for the pressure comparison calibration of ${¼}^{\prime \prime }$${\mathrm{Mic}}_{\mathrm{test},\mathrm{WS}3}$ against ${½}^{\prime \prime }$${\mathrm{Mic}}_{\mathrm{ref},\mathrm{LS}2}$, and the correction values are provided by IEC 61094-5 [20]. In this specific calibration, sound field corrections become significant for frequencies above 5 kHz, reaching absolute values greater than 1.4 dB at 20 kHz.

The model assumes a specific set of conditions related to the microphones’ geometry and acoustic impedance. These conditions are integral to the calibration procedure, as the model calculates the pressure distribution and the subsequent sound field corrections for the microphones based on the physical properties and boundary conditions of the system. For microphones with identical geometries and similar acoustic impedance properties, the analytical model remains highly effective. However, it should be noted that the application of this model to microphones that deviate from these assumptions—especially for microphones with different geometries or distinct impedance characteristics—can lead to inaccuracies, particularly when performing pressure comparison calibrations.

For example, when dealing with MEMS microphones, which are characterized by their miniaturized structures and unique impedance properties, the assumptions underlying the analytical model may no longer hold true. MEMS microphones are often subjected to different acoustic interactions due to their small size and specific design features, such as the top-port or bottom-port configuration. Consequently, pressure comparison calibration using the analytical model may yield unreliable results in such cases.

To overcome these limitations, a more sophisticated approach is required, especially when dealing with MEMS microphones or other types of specialized sensors. In these cases, a numerical approach such as FEM is recommended, as discussed below.

### 2.2. Numerical Solution

FEM is a numerical approach to approximate solutions to partial differential equations. This method is extensively employed in engineering to address problems where the analytical solution is either unknown or overly complex to develop. One of its primary advantages is its flexibility in handling complex geometries. The solution domain $\Omega \subset R^{n}$ is approximated by a union of smaller disjoint subdomains $\cup \Omega^{\left(e\right)}$, known as finite elements, with the problem’s nodes located at the vertices of these elements [26,27,28].

In this section, key considerations for modeling the sound field between two microphones using the FEM will be discussed. The numerical modeling of the sound field can provide an accurate description of the acoustic pressure distribution in the narrow gap between the microphones, addressing the variety of geometries and installation configurations, as well as the size dissimilarity between the reference microphone and the point-like sensing surface of MEMS microphones.

Sound field corrections are calculated based on the acoustic pressure distribution over the sensing surfaces of both the ${\mathrm{Mic}}_{\mathrm{ref}}$ and ${\mathrm{Mic}}_{\mathrm{test}}$. The spatial distribution of the acoustic pressure *p* can be derived from the Helmholtz equation [29] in Equation (3).

(3) $$\Delta p+k^{2}p=0$$

where k=ω/c is the wave number, ω is the angular frequency, and *c* is the speed of sound in air. To solve Equation (3), FEM can be used, considering the following boundary conditions:

(i) For rigid boundaries characterized by infinite acoustic impedance,
  (4) ∇p·n=0;
(ii) For vibrating boundaries with finite acoustic impedance modulus *Z*,
  (5) $$\nabla p\;\cdot \;n=-j\frac{\omega \rho p}{|Z|};$$
(iii) For sound source boundaries with known distribution of acoustic particle velocity *v*,
  (6) ∇p·n=−jωρv,
  being *n* the unit vector normal to the specific boundary surface and ρ the air density.

The numerical approach to solving the Helmholtz equation enables the handling of complex geometries, while also accounting for the influences of Helmholtz resonant cavities, such as those formed by the MEMS cavity and the pressure hole, as found in typical bottom-port or top-port MEMS microphones mounted on evaluation Printed Circuit Boards (PCBs) [30]. In this regard, it is important to preliminarily decide whether or not to consider the influence of Helmholtz resonant cavities when evaluating sound field corrections. Depending on this choice, the correction values may vary, leading to different sensitivity values, particularly at frequencies above 4 kHz. Furthermore, in most cases, the actual dimensions of MEMS cavities and pressure holes are unknown, meaning that the uncertainty associated with sound field corrections may significantly increase if the influence of Helmholtz resonant cavities is taken into account.

**Figure 2:**
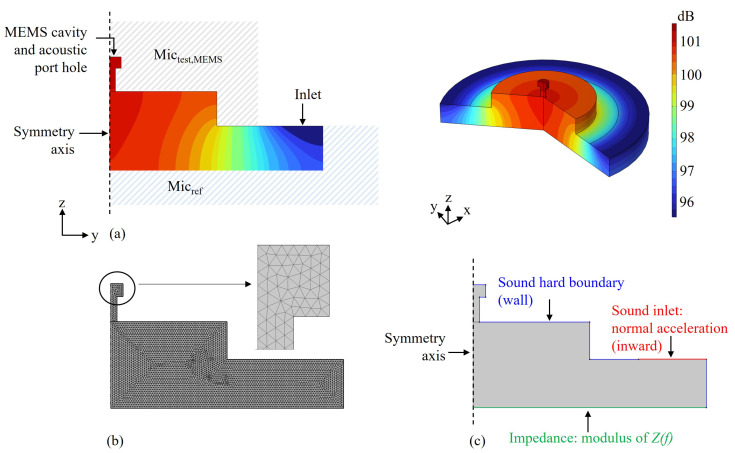
(**a**) Example of numerical simulation of the acoustic field for pressure comparison calibration of a MEMS microphone in 2D and 3D for a frequency of 8 kHz; (**b**) meshing; (**c**) boundary conditions.

The FEM analyses presented in this work are based on the description of finite acoustic impedance boundaries using a lumped-parameter approach. The geometry, frequencies of interest and meshing, and boundary conditions are described below:

- Geometry: The proposed design exhibits a symmetry of revolution, and it can be treated as an axisymmetric problem. This allows a 3D problem to be simplified into a 2D axisymmetric problem. The response of an acoustic system to a harmonic excitation is simulated.
  The simulation of acoustic fields in the pressure comparison calibration of MEMS microphones can be effectively focused on analyzing coupling configurations, where the assumption of a circular axisymmetric domain holds. These types of set-ups are recommended for obtaining repeatable results due to the good reproducibility of the coaxial alignment of microphones and the sound source, and can be simulated using 2D cylindrical coordinates. Figure 2a shows an example of the computational domain for this kind of pressure comparison set-up.
  The diaphragm and typical structure of reference condenser microphones can be modeled as dynamic systems with equivalent mass, compliance, and resistance [31,32], or alternatively, as equivalent volume, resonance frequency, and loss factor.
- Frequencies of interest and meshing: A mesh with a maximum element size of λ/30 is used, where λ is the wavelength corresponding to the maximum simulation frequency ($f_{max}=$ 30 kHz). Each mesh element has a triangular shape to efficiently cover the entire structure using an extremely fine mesh generator. Thus, the number of mesh elements was 7618 with a total mesh area of 25.55 ${\mathrm{mm}}^{2}$. An example of this is shown in Figure 2b.
- Boundary conditions:
  - Axial symmetry: This is a default boundary condition used to obtain such symmetry. It is set on the longitudinal axis of the configuration.
  - Sound hard boundary: It is applied to the non-vibrating parts of microphones to prevent the transfer of energy into the resonant cavities.
  - Impedance: It is applied to vibrating boundaries, like the microphones’ diaphragms, whose acoustic impedance is a function of the specific characteristic parameters of the microphones (equivalent volume, resonance frequency, and loss factor), environmental conditions and frequency.
  - Normal acceleration: A specific acceleration is applied in the direction perpendicular to the face of the microphones to analyze the acoustic pressure derived directly from the applied acceleration.
  Figure 2c shows these boundary conditions used in the FEM simulations.

In addition, the structure and characteristic parameters of the microphones used are described in detail in Appendix A.

**Figure 3:**
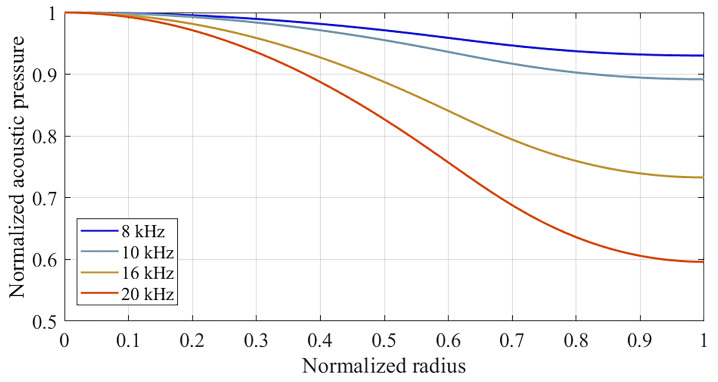
Frequency dependence of the radial distribution of acoustic pressure at the reference microphone diaphragm, as obtained from FEM simulation for a specific pressure comparison configuration.

Figure 2a shows an example of the Sound Pressure Level (SPL) distribution for a given pressure comparison configuration. The mean pressures along the radii of the microphones’ sensing surfaces can be calculated from the acoustic pressure distribution over the computational domain, optionally considering the theoretical radial distribution of the microphone’s sensitivity as the weighing factor for the integral mean. Figure 3 shows an example of the radial distribution of the normalized acoustic pressure over the reference microphone diaphragm, as obtained through numerical simulation of the acoustic field at different frequencies for a specific pressure comparison configuration.

The radial distribution of the acoustic pressure at the microphones’ sensing surfaces can also be used to evaluate the uncertainty contribution associated with potential axial misalignment between the test and reference microphones. Specifically, this contribution can be assessed from the ratio between the mean acoustic pressure at the possible off-axis position of the test microphone and the mean acoustic pressure at perfect coaxial alignment.

To provide a reliable estimation of sound field corrections and quantify their accuracy, the numerical modeling of acoustic fields must first be validated. Validation has been carried out for the specific coupling configuration where an analytical solution of the acoustic field is available for evaluating sound field corrections [29], i.e., the one discussed in Section 2.1. For this pressure comparison configuration, the analytical estimation of corrections was experimentally validated by comparing the results of pressure comparison calibration with those obtained from primary reciprocity calibration [24].

## 3. Calibration of MEMS Microphones by the Active Coupler

An experimental set-up for the pressure comparison calibration of a digital MEMS microphone inside an anechoic chamber was previously discussed in [33]. Another practical implementation of the pressure comparison method, relying on simultaneous acoustic excitation provided by the active coupler, can be applied to the calibration of analog MEMS microphones [22]. Regarding this practical implementation of the comparison method, the experimental set-up and the measurement solutions adopted to compensate for the effects of sound field asymmetries inside the active coupler will be detailed hereinafter.

### 3.1. Experimental Set-Up

Among the commonly used comparison calibration techniques for pressure couplers, the solution based on the simultaneous excitation of ${\mathrm{Mic}}_{\mathrm{ref}}$ and ${\mathrm{Mic}}_{\mathrm{test}}$ by the active coupler Brüel & Kjær WA0817 has been selected. This approach is particularly promising for MEMS microphone calibration as it provides a good trade-off between the speed, cost-effectiveness, and comprehensive determination of the frequency response over a wide frequency range.

The ${\mathrm{Mic}}_{\mathrm{test},\mathrm{MEMS}}$, preliminarily mounted in a custom-made cartridge, and the ${\mathrm{Mic}}_{\mathrm{ref},\mathrm{LS}2}$ were both placed into the active coupler and simultaneously exposed to the same sound field. The specific design of the cartridge, in which the ${\mathrm{Mic}}_{\mathrm{test},\mathrm{MEMS}}$ is housed, creates a pressure cavity inside the active coupler similar to those formed by typical condenser microphones calibrated using this technique. This ensures the proper nominal operation of the active coupler and is expected to improve the reliability and accuracy of the numerical evaluation of the acoustic field, due to the simple geometrical layout.

Stationary sinusoidal signals at exact mid-band frequencies for octave-bands from 501.19 Hz to 3981.07 Hz, and for one-third-octave-bands from 5011.87 Hz to 15,848.93 Hz, with the additional frequencies of 11,220.18 Hz, 14,125.38 Hz, and 17,782.79 Hz, were provided to the active coupler through the signal generator, averaging the output signals of the microphones over a time considerably longer than their period T=1/f. In this way, the phase difference between the microphones’ signals did not require a phase correction of the field. However, in the presence of a complex signal spectrum, such a phase shift must be considered, evaluated, and corrected if necessary.

With respect to the characteristics of the active coupler, the frequency response of the loudspeaker as specified by the manufacturer was considered in order to approximately equalize the SPL in the cavity between the microphones inside the active coupler over the frequency range of interest. Specifically, the amplitude of sinusoidal signals sent by the signal generator to the audio amplifier connected to the active coupler was 1 V rms for frequencies below 2 kHz and above 16 kHz, and from 0.25 V rms to 0.7 V rms in the frequency range from 4 kHz to 16 kHz, with a minimum amplitude of 0.25 V rms at 8 kHz. This ensured an SPL of approximately 90 dB as generated by the active coupler in the frequency range from 501.19 Hz to 17,782.79 Hz.

**Figure 4:**
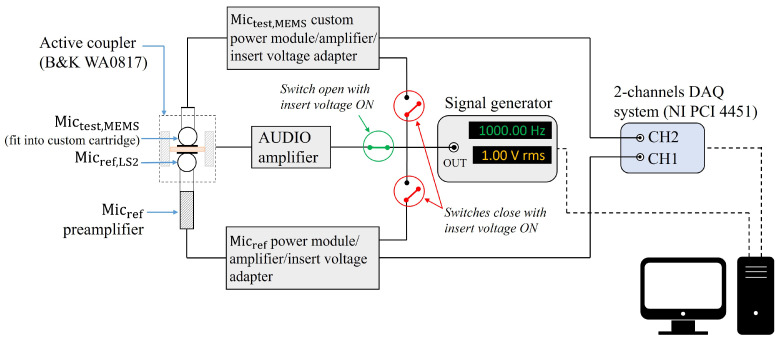
Sketch of the experimental set-up for pressure comparison calibration of the ${\mathrm{Mic}}_{\mathrm{test},\mathrm{MEMS}}$ by means of the active coupler.

Regarding the electrical part of the system, the MEMS microphone was connected to a custom circuit designed for power supply, signal conditioning, and the application of the insert voltage technique, analogous to the amplification and conditioning system connected to the reference condenser microphone. The ability to implement the insert voltage technique for both microphones is crucial, as it allows for the compensation of gains between the two measurement chains, significantly reducing calibration uncertainty. The output signals from the two measurement chains were measured simultaneously by a two-channel Data Acquisition (DAQ) system (NI PCI 4451), which supports frequency-selective techniques, such as Fast Fourier Transform (FFT) analysis, to improve the signal-to-noise ratio and minimize distortion effects on the measurement. The measurement system and the configuration of the switches for the application of the insert voltage technique are illustrated in Figure 4.

**Figure 5:**
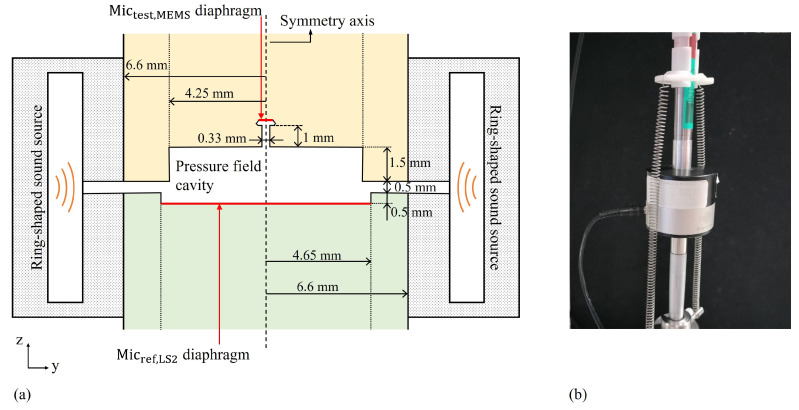
(**a**) Zoom of the longitudinal section of the B&K WA0817 active coupler sketched in Figure 4, when coupled to the ${\mathrm{Mic}}_{\mathrm{test},\mathrm{MEMS}}$ mounted in the custom cartridge, and the ${\mathrm{Mic}}_{\mathrm{ref},\mathrm{LS}2}$; (**b**) a photo of both microphones fit into the active coupler during calibration.

The dimensional parameters of the internal cavities considered for the FEM analysis were obtained from the data available from the manufacturers of the microphones and the active coupler, and from the measured dimensions of the special MEMS cartridge. The dimensional characteristics of the pressure field cavity of the active coupler are shown in Figure 5a, together with a sketch of the longitudinal section of the cylindrical active coupler, including the details of the volumes related to the microphones, the annular cavity of the ring-shaped speaker, and the air separation gap in which the acoustic field is simulated. It is worth noting that the uncertainty associated with these dimensional parameters can contribute significantly to the uncertainty of the sound field corrections. For this purpose, the FEM simulation can be an effective tool to evaluate such a contribution by changing the value of the dimensional parameters according to their probability distribution and solving the corresponding acoustic field. Figure 5b shows a picture of both the reference and MEMS microphones fit into the active coupler during calibration.

It is important to note that the influence of the Helmholtz resonance due to the MEMS microphone cavity and pressure hole should only be considered if the sensitivity to be measured is defined as the ratio between the microphone’s open-circuit voltage and the actual acoustic pressure over a specific measurement surface (e.g., the surface of the acoustic port on the PCB frontside). On the other hand, if the sensitivity to be measured is defined based on the undisturbed sound field for the evaluation of acoustic pressure, the Helmholtz resonant cavity of the MEMS microphone should not be simulated, and the MEMS sensing surface should be assumed to be flush-mounted on the PCB acoustic port. This configuration is referred to hereinafter as the “ideal configuration”.

### 3.2. Compensation of Sound Field Asymmetries

**Figure 6:**
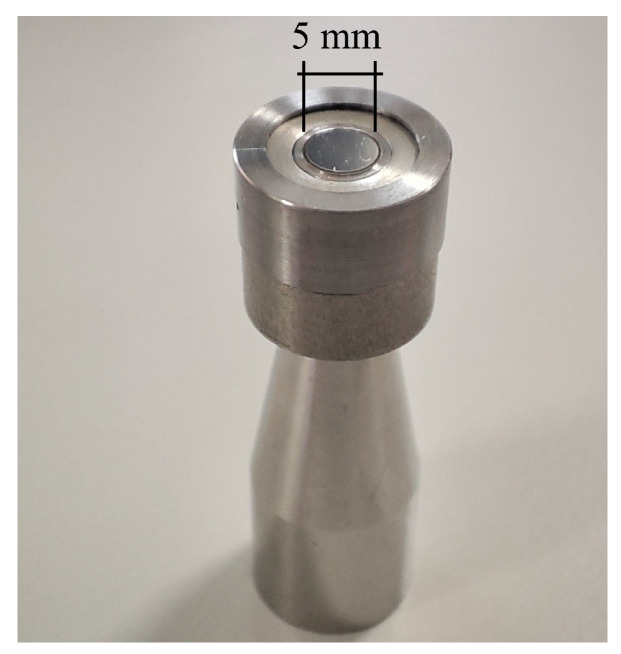
Special ${½}^{\prime \prime }$ adapter for the calibration of the WS3 microphone by the active coupler.

An important point of discussion concerns the reliability of the assumption of a 2D axisymmetric domain for the numerical solution of the acoustic field. In fact, the condition of perfect circular symmetry of the sound source is difficult to achieve in practice, particularly at high frequencies, and asymmetric acoustic pressure distributions are likely to occur in the air gap between the microphones. An experimental procedure was conducted to assess the contribution of asymmetric sound fields inside the active coupler. The method involved using both a reference LS2 microphone and a reference WS3 microphone to calibrate the same MEMS test microphone. The WS3 microphone was installed without its protective grid into the special adapter shown in Figure 6, which ensured that the WS3 diaphragm is flush-mounted in a structure that mimics the layout of an LS2 microphone. With this special adapter, the WS3 microphone was calibrated, as a test microphone, against the LS2 reference microphone using the active coupler.

By combining the three measurement models described by Equation (2) for the three comparison calibrations, namely (i) MEMS vs. LS2, (ii) MEMS vs. WS3, and (iii) WS3 vs. LS2, it is possible to obtain the following Equation (7), which relates the voltage ratios $L_{V,j}$, in dB, to the actual values of sound field corrections $\delta_{j}$, with the subscript j=1,2,3 corresponding to cases (i), (ii), and (iii), respectively.

(7) $$\left(L_{V,1}+\delta_{1}\right)-\left(L_{V,2}+\delta_{2}\right)-\left(L_{V,3}+\delta_{3}\right)=0$$

Considering the measured values of the voltage ratios and the numerical estimates of sound field corrections $\delta_{FEM,j}$ under 2D axisymmetric assumptions, Equation (8) provides the overall error ε in the estimation of sound field corrections, due to the actual asymmetries of the acoustic field inside the active coupler.

(8) $$\varepsilon =\left(L_{V,1}+\delta_{FEM,1}\right)-\left(L_{V,2}+\delta_{FEM,2}\right)-\left(L_{V,3}+\delta_{FEM,3}\right)$$

Such an overall error can be assumed to be distributed among the three different calibrations (MEMS vs. LS2, MEMS vs. WS3, and WS3 vs. LS2) according to the specific value of the sound field corrections obtained from numerical simulations under 2D axisymmetric assumptions. The actual values of sound field corrections $\delta_{j}$ were calculated from the numerical values $\delta_{FEM,j}$ using the following Equation (9):

(9) $$\delta_{j}=\delta_{FEM,j}+\varepsilon \frac{r_{j}}{2r_{j}-1}$$

where $r_{j}$ is the numerical sound field correction ratio, defined as follows:

(10) $$r_{j}=\frac{\delta_{FEM,j}}{\sum_{i=1}^{3}\delta_{FEM,i}}$$

An important issue to consider is the potential instability of the acoustic field across the three different calibrations mentioned above. In fact, the active coupler is not expected to produce exactly the same SPL inside the cavity between the microphones, even though the same signal is provided by the function generator. To compensate for the potential instability of the acoustic field, the measurement of the voltage ratios at low frequencies (below 1 kHz) can be exploited, where the sound field corrections are expected to be negligible due to the spatial uniformity of the acoustic pressure inside the active coupler. Specifically, the measurements of the voltage ratios can be uniformly corrected by a quantity $\varphi =(L_{V,1}-L_{V,2}-L_{V,3})/3$, where the voltage ratios $L_{V,j}$ are those measured at frequencies lower than 1 kHz. The steps of the above-detailed experimental procedure are summarized in Appendix B.

## 4. Results and Discussion

The frequency response of the MEMS microphone sensitivity in pressure-field conditions was obtained by evaluating the sound field correction values for the “ideal configuration”, i.e., the scenario where the acoustic field is not influenced by the resonant cavity of the MEMS microphone. The sound field corrections obtained for this configuration can significantly differ from the correction values calculated when considering the influence of the resonant cavity of the MEMS microphone on the sound field.

**Figure 7:**
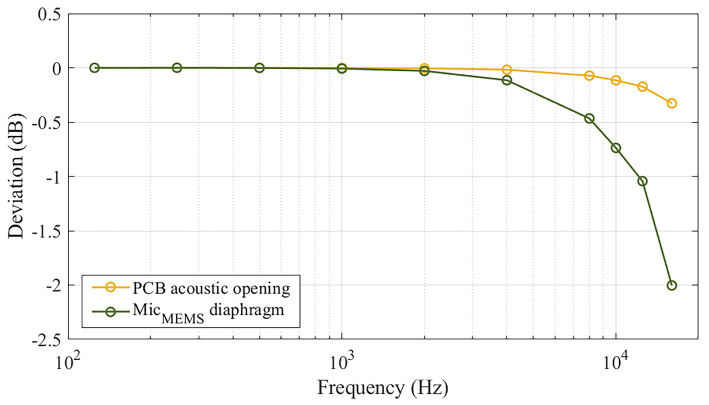
Deviation of sound field corrections with respect to the “ideal configuration”, depending on the definition of the acoustic pressure measurement surface for the MEMS microphone.

Figure 7 illustrates this deviation, obtained through an FEM simulation, as a function of frequency and the location at which the acoustic pressure is evaluated, e.g., on the MEMS diaphragm or at the circular opening of the acoustic port hole on the PCB frontside. For the case in which the sound pressure was evaluated at the PCB opening of the acoustic port hole, the deviation with respect to the “ideal configuration” revealed to be negligible up to 4 kHz, reaching a value of about −0.3 dB at 16 kHz. Conversely, for the situation in which the acoustic pressure was assessed at the MEMS microphone’s diaphragm, the deviation was markedly more pronounced; indeed, the deviation started to be significant even at 4 kHz, reducing to a value of −2 dB at 16 kHz. This is important to be considered for the evaluation of the pressure sensitivity of the MEMS microphone, as the values of the sound field corrections change considerably. This particularly occurs at high frequencies, based on the defined measurement location of the acoustic pressure, thus leading to significantly different calibration results.

Table 1 summarizes the measured voltage ratios $L_{V,j}$ corrected for sound source instability across the three calibrations: MEMS vs. LS2, MEMS vs. WS3, and WS3 vs. LS2. It is important to note that a 20 dB amplification gain was applied only to the WS3 measurement chain due to the lower sensitivity of the WS3 microphone. Additionally, insert voltage preamplifiers were used for both the LS2 and WS3 microphones to enable the application of the insert voltage technique, as described in the experimental set-up outlined in Figure 4.

In the experiments, the instability observed in the acoustic field was exclusively related to the differences in acoustic pressure between the different calibration configurations (MEMS vs. LS2, MEMS vs. WS3, and WS3 vs. LS2). The signal used was always stationary, but the loudspeaker of the active coupler could have generated a different SPL in the three calibration configurations, which would require a correction if the microphone sensitivity varies with the SPL, as described in Section 3.2. Such a correction was evaluated from the low frequency values of the voltage ratio measurements, and was observed to be around 0.02 dB. Even within the same calibration configuration, the acoustic field could exhibit instabilities, suggesting the need to evaluate a phase correction. However, since the stability of the microphone output signals was monitored during the calibrations and relative standard deviations of less than 10 ppm were observed, the effect of the phase is considered to have a minimal contribution to the uncertainty and thus can be neglected.

The acoustic field inside the active coupler was characterized by an approximately flat spectrum over the frequency range of interest, from 501.19 Hz to 17,782.79 Hz, thanks to the equalization of the SPL according to the frequency response of the loudspeaker, as described in Section 3.1. In this way, the pressure field cavity between the microphones was excited approximately with the same sound energy over the frequency interval under investigation, with the specific frequency-dependent spatial distribution of the acoustic pressure.

It is worth observing that the signal-to-noise ratio at each frequency and experimental configuration was always higher than 40 dB, preserving the measurement accuracy from the influence of the background noise.

Table 2 reports the differences between the voltage gains ΔG of the reference and MEMS microphone measurement chains, as obtained for the MEMS vs. LS2 and MEMS vs. WS3 calibrations, through the application of the insert voltage technique. The insert voltage provided by the function generator was 20 mV RMS.

**Figure 8:**
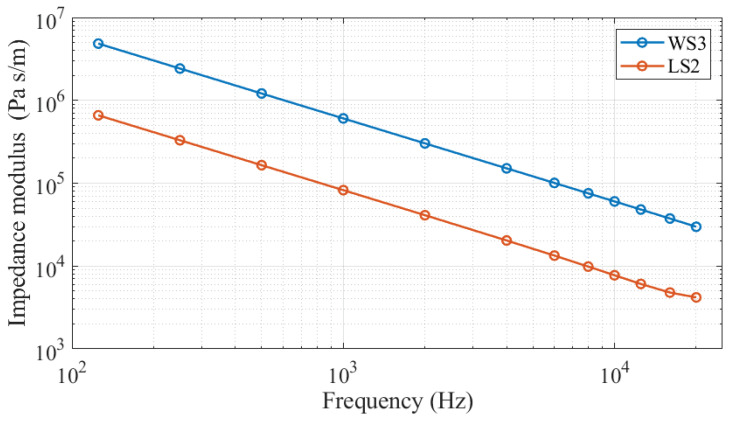
Frequency dependence of the acoustic impedance modulus for the LS2 and WS3 microphones used in the experiments and characterized by the sizes and lumped-parameters reported in Appendix A.

For the LS2 and WS3 microphones, the FEM simulation of the acoustic field requires the knowledge of the corresponding characteristic lumped-parameters to describe their acoustic impedance. In particular, with the values reported in Appendix A for diaphragm radius, resonance frequency, equivalent volume, and loss factor, the complex acoustic impedances of LS2 and WS3 microphones used in the experiments can be evaluated, and the resulting frequency dependence of their modulus is plotted in Figure 8. These acoustic impedance moduli, expressed as a function of frequency, are used for the FEM solution of the acoustic field in the narrow space between microphones inside the active coupler. Regarding the MEMS microphone, a sound hard boundary condition, representing an infinite acoustic impedance, was assumed. This can be regarded as a reasonable assumption, due to the miniaturized size of the sensing element of the MEMS microphone, and to its negligible contribution to the transfer of energy into the pressure cavity.

**Figure 9:**
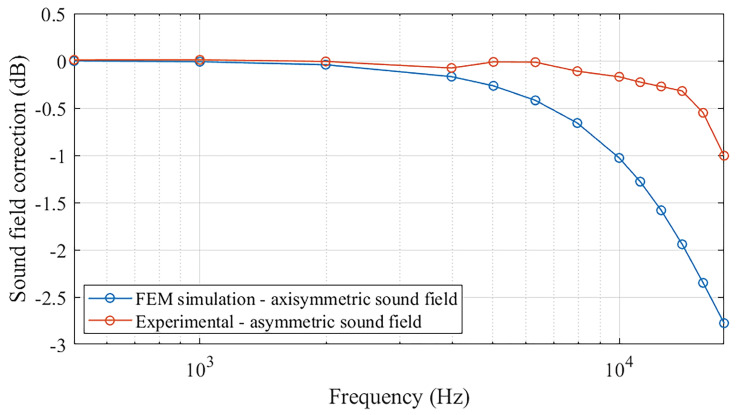
Frequency dependence of sound field correction values for MEMS microphone calibration by the active coupler, with and without errors compensation for real asymmetric sound field.

Figure 9 shows the frequency dependence of the actual sound field correction values obtained through the experimental method, based on the measurement results reported in Table 1. The actual sound field correction values, related to the MEMS vs. LS2 calibration, are compared with the numerical simulation results under ideal axisymmetric assumptions.

Interestingly, the active coupler used in this study proved to produce an acoustic field in which the sound field correction values were not as prominent as the numerical simulations predicted. This behavior is possibly attributable to the complex non-uniformities of the acoustic pressure occurring inside the specific active coupler, mainly arising from the asymmetric radiation of the sound source and the potentially slightly asymmetrical configuration of the cavity formed between the reference and MEMS microphones.

**Figure 10:**
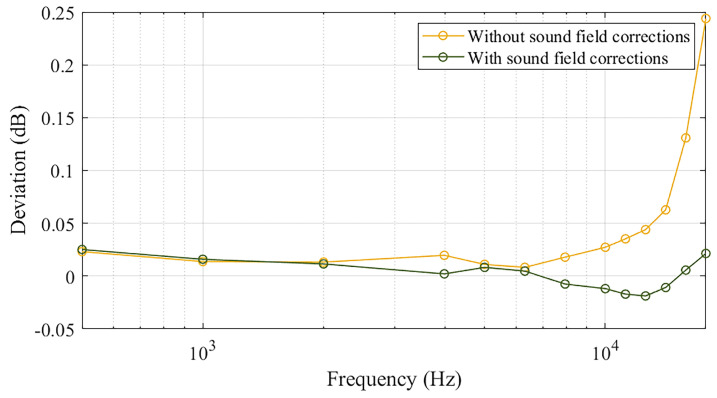
Deviation between the MEMS sensitivity levels obtained using the ${\mathrm{Mic}}_{\mathrm{ref},\mathrm{LS}2}$ and the ${\mathrm{Mic}}_{\mathrm{ref},\mathrm{WS}3}$ in the active coupler; deviations are plotted as a function of frequency with and without the application of the sound field corrections.

Finally, Figure 10 represents a proof of the validation of the proposed calibration procedure. In fact, the deviation between the calibration results of the MEMS microphone sensitivity, obtained by using two different reference microphones, the LS2 and the WS3, was observed to be within ±0.03 dB over the whole frequency range from 501.19 Hz to 17,782.79 Hz. The absolute value of such a deviation was lower than the measurement uncertainty associated with the sensitivity level of the LS2 microphone, obtained by the primary reciprocity calibration method, which is equal to 0.05 dB from 500 Hz to 8 kHz, 0.08 dB up to 10 kHz, 0.10 dB up to 12.5 kHz, and 0.15 dB up to 20 kHz. This demonstrated the effectiveness of the proposed numerical and experimental procedure for the accurate evaluation of the sound field corrections.

Concerning the environmental conditions during the tests, the measurement results reported above were obtained at static pressure ranging between 99.98 kPa and 100.35 kPa, air temperature between 22.9 °C and 24.2 °C, and relative humidity ranging between 30% and 33%.

The main source of uncertainty in the estimation of sound field corrections is the lack of knowledge about the actual distribution of acoustic pressure in the narrow gap between the microphones. This uncertainty arises primarily from the characteristics of the sound source and its ability to generate a uniform and circular-symmetric acoustic excitation.

The experimental procedure for evaluating the effects of asymmetric sound fields assumes that error compensations for real acoustic fields are directly proportional to the sound field corrections calculated under ideal axisymmetric conditions. However, it is also assumed, as a limit case, that asymmetric fields may produce null sound field correction values, meaning that the reference and test microphones may experience the same average acoustic pressure, even at high frequencies. For this reason, the uncertainties associated with sound field corrections can be prudently assumed to follow a uniform probability distribution.

## 5. Conclusions

This paper addressed the metrological aspects of MEMS microphones calibration by the secondary pressure comparison method, focusing on overcoming the limitations of existing models and the challenges of high-frequency measurements. Due to their miniaturized size and non-standard geometries, the effect of non-uniform acoustic pressure distributions in the narrow spaces between the reference and test microphones in pressure comparison calibration set-ups needs to be carefully handled and compensated, especially at high frequencies. In addition, traditional analytical models suitable for standard laboratory condenser microphones do not reflect the unique characteristics of MEMS.

To address these issues, this paper proposed a numerical and experimental approach based on FEM to resolve the acoustic field in the complex pressure cavities inherent to MEMS microphone calibration. By using a lumped-parameter approach to model the finite acoustic impedance boundaries, the interactions between the microphones and their environment can be accurately simulated. However, this approach faces significant challenges when applied to non-ideal geometries and at high frequencies, where the acoustic behavior becomes very sensitive to small deviations in the physical design.

The results presented in this paper provide a solid foundation for extending the pressure comparison method to MEMS microphones, potentially enabling their calibration over a wider frequency range with reliable sound field correction assessments. However, as research continues, the challenges outlined here, particularly those related to frequency-dependent corrections, environmental factors, and limitations of existing models, must be addressed to ensure that the pressure comparison method can be effectively applied to a wide range of microphone types. Further developments towards the improvement of the time and cost effectiveness of the measurement procedure are expected, as far as the use of complex signal spectra combined with techniques for the phase correction of the acoustic field is concerned. By refining numerical models, improving experimental set-ups, and incorporating advanced computational techniques, future work will help to advance the field of acoustic MEMS metrology and drive the innovation in the calibration of next-generation microphones and sensors.

## Figures and Tables

**Table 1:** Voltage ratio measurements expressed in dB as a function of frequency.

Frequency (Hz)	$L_{V,1}$ (dB)	$L_{V,2}$ (dB)	$L_{V,3}$ (dB)
501.19	−1.76	0.01	−1.78
1000.00	−1.70	0.07	−1.77
1995.26	−1.70	0.05	−1.75
3981.07	−1.98	−0.29	−1.67
5011.87	−2.24	−0.61	−1.62
6309.57	−2.70	−1.13	−1.56
7943.28	−3.49	−2.00	−1.46
10,000.00	−4.96	−3.53	−1.39
11,220.18	−6.15	−4.72	−1.38
12,589.25	−7.85	−6.35	−1.44
14,125.38	−10.17	−8.47	−1.62
15,848.93	−12.30	−10.14	−2.03
17,782.79	−11.59	−8.59	−2.77

**Table 2:** Gain difference of the two measurement chains expressed in dB as a function of frequency.

Frequency (Hz)	ΔG (MEMS vs. LS2) (dB)	ΔG (MEMS vs. WS3) (dB)
501.19	−0.07	19.89
1000.00	−0.07	19.90
1995.26	−0.07	19.90
3981.07	−0.05	19.92
5011.87	−0.03	19.94
6309.57	0.00	19.96
7943.28	0.04	20.01
10,000.00	0.11	20.07
11,220.18	0.15	20.12
12,589.25	0.21	20.18
14,125.38	0.29	20.25
15,848.93	0.38	20.34
17,782.79	0.50	20.46

## Data Availability

The data presented in this study can be made available upon request from the corresponding authors.

## Appendix A

The structure and characteristic parameters of microphones determine their performance and suitability for various applications. Below are the dimensions and acoustic properties of the microphones used in the experimental measurements.

Table A1 summarizes the geometric and acoustic characteristics of the LS2 and WS3 microphones.

**Table A1:** Geometric and acoustic characteristics of the LS2 and WS3 microphones.

		Mic LS2	WS3 (Fit into Special Adapter)
Diaphragm radius	*a*	4.65 mm	2.065 mm
Outer radius	$a_{out}$	6.6 mm	2.975 mm
Front cavity depth	*I*	0.5 mm	0.5 mm
Equivalent volume	$V_{eq}$	9.2 ${\mathrm{mm}}^{3}$	0.25 ${\mathrm{mm}}^{3}$
Resonance frequency	$f_{0}$	20.6 kHz	100 kHz
Loss factor	*d*	1.11	1.11
Sensitivity level at 250 Hz	*S*	−38.2 dB (re 1 V/Pa)	−56.3 dB (re 1 V/Pa)

Table A2 summarizes the geometric and acoustic characteristics of the MEMS microphone fit into custom cartridge.

**Table A2:** Characteristics of the MEMS microphone fit into custom cartridge.

MEMS (Fit into Custom Cartridge)		
Inner cartridge radius	$b_{in}$	4.25 mm
Outer cartridge radius	$b_{out}$	6.6 mm
Front cavity depth	*I*	1.5 mm
Acoustic port hole diameter	ϕ	0.33 mm
Acoustic port hole depth	*h*	1 mm
Sensitivity level (100 Hz–10 kHz)	*S*	−38 ± 3 dB (re 1 V/Pa)

## Appendix B

Below is a detailed breakdown of the experimental method that was designed and implemented to evaluate the effect of such complex asymmetric non-uniformities within a specific active coupler:

1. Measurement of the voltage ratios of MEMS vs. LS2 and MEMS vs. WS3 comparison configurations, where an LS2 (1/$2^{\prime \prime }$ diameter) and a WS3 (1/$4^{\prime \prime }$ diameter) microphone were used as reference to calibrate the same MEMS microphone.
2. Measurement of the voltage ratio for the WS3 vs. LS2 comparison configuration, where the LS2 microphone was considered as the reference one.
3. The product of the measured voltage ratios is equal to the product of the ratios of the actual acoustic pressures sensed by MEMS, LS2 and WS3 microphones in the three comparison configurations.
4. The same product of the ratios of actual acoustic pressures was evaluated by the FEM simulation and compared with the value obtained experimentally; the deviation from the experimental value was used to correct the pressure ratio estimates based on their FEM values.

## References

[1] He Y., Yu A., Liu X., Wang Y., Wang Y., Chi M.H., Lou J.J.C., Chen C.Z. (2024). Micro-Electro-Mechanical Systems (MEMS). Handbook of Integrated Circuit Industry.

[2] Gemelli A., Tambussi M., Fusetto S., Aprile A., Moisello E., Bonizzoni E., Malcovati P. (2023). Recent trends in structures and interfaces of MEMS transducers for audio applications: A review. Micromachines.

[3] Shah M.A., Shah I.A., Lee D.G., Hur S. (2019). Design approaches of MEMS microphones for enhanced performance. J. Sens..

[4] Marek J., Gómez U.M., Hoefflinger B. (2011). MEMS (Micro-Electro-Mechanical Systems) for automotive and consumer electronics. Chips 2020: A Guide to the Future of Nanoelectronics.

[5] Fawzy A., Magdy A., Hossam A. (2022). A piezoelectric MEMS microphone optimizer platform. Alex. Eng. J..

[6] Mukherjee S., Suleman S., Pilloton R., Narang J., Rani K. (2022). State of the art in smart portable, wearable, ingestible and implantable devices for health status monitoring and disease management. Sensors.

[7] Tiete J., Domínguez F., da Silva B., Touhafi A., Steenhaut K., Uttamchandani D. (2017). MEMS microphones for wireless applications. Wireless MEMS Networks and Applications.

[8] Judy J.W. (2001). Microelectromechanical systems (MEMS): Fabrication, design and applications. Smart Mater. Struct..

[9] Quintero G., Balastegui A., Romeu J. (2019). A low-cost noise measurement device for noise mapping based on mobile sampling. Measurement.

[10] Picaut J., Can A., Fortin N., Ardouin J., Lagrange M. (2020). Low-cost sensors for urban noise monitoring networks—A literature review. Sensors.

[11] Izquierdo A., Del Val L., Villacorta J.J., Zhen W., Scherer S., Fang Z. (2020). Feasibility of discriminating UAV propellers noise from distress signals to locate people in enclosed environments using MEMS microphone arrays. Sensors.

[12] Tiete J., Domínguez F., Da Silva B., Segers L., Steenhaut K., Touhafi A. (2014). SoundCompass: A distributed MEMS microphone array-based sensor for sound source localization. Sensors.

[13] Ramamonjy A., Bavu E., Garcia A., Hengy S. Source localization and identification with a compact array of digital mems microphones. Proceedings of the 25th International Congress on Sound and Vibration (ICSV25).

[14] Rahaman A., Kim B. (2020). Sound source localization by *Ormia ochracea* inspired low–noise piezoelectric MEMS directional microphone. Sci. Rep..

[15] Ishfaque A., Kim B. (2022). Real-time sound source localization in robots using fly *Ormia ochracea* inspired MEMS directional microphone. IEEE Sens. Lett..

[16] Kalra M., Kumar S., Das B. (2019). Moving ground target detection with seismic signal using smooth pseudo Wigner–Ville distribution. IEEE Trans. Instrum. Meas..

[17] Liu H., Shi J., Huang J., Zhou Q., Wei S., Li B., Yuan X. (2019). Single-mode wild area surveillance sensor with ultra-low power design based on microphone array. IEEE Access.

[18] Gazivoda M., Bilas V. (2022). Always-on sparse event wake-up detectors: A Review. IEEE Sens. J..

[19] Svatos J., Holub J. (2023). Impulse acoustic event detection, classification, and localization system. IEEE Trans. Instrum. Meas..

[20] (2016). Electroacoustics—Measurement Microphones—Part 5: Methods for Pressure Calibration of Working Standard Microphones by Comparison.

[21] Au A.C., Lam B.H., Chau Y. Calibration of microphones in accordance with the International Standard IEC 61094-5:2016. Proceedings of the INTER-NOISE and NOISE-CON Congress and Conference.

[22] Saba F., Durando G., Paesante D., Corallo M., Schiavi A., Prato A. An experimental setup for the metrological characterization of MEMS microphones. Proceedings of the 24th International Congress on Acoustics (ICA 2022)-A05: Electro-Acoustics.

[23] Blanford T.E., Rusk Z. Summary of “Microphones: Design, Development, and Characterization”. Proceedings of the Meetings on Acoustics.

[24] Jarvis D., Watkins S.A. (1997). Methods for Determining the Pressure Sensitivity of IEC type WS3 Measurement Microphones. Technical Report, NPL Report. CIRA(EXT)022. https://eprintspublications.npl.co.uk/701/.

[25] Barham R., Barrera-Figueroa S., Avison J.E. (2014). Secondary pressure calibration of measurement microphones. Metrologia.

[26] Hughes T.J. (2012). The Finite Element Method: Linear Static and Dynamic Finite Element Analysis.

[27] Fernández-Garrido A., Campo-Valera M., Abdo-Sánchez E., Picó R., Garcia-Sanchez A.J., Asorey-Cacheda R. (2025). Parametric Study and Experimental Validation of Acoustic Leaky Wave Antenna in Spatial Localization. IEEE Access.

[28] Campo-Valera M., Asorey-Cacheda R., Rodríguez-Rodríguez I., Villó-Pérez I. (2023). Characterization of a piezoelectric acoustic sensor fabricated for low-frequency applications: A comparative study of three methods. Sensors.

[29] Saba F., Paesante D., Durando G., Corallo M. Sound field corrections for secondary pressure calibration of mems microphones. Proceedings of the 10th Convention of the European Acoustics Association—Forum Acusticum 2023.

[30] STMicroelectronics (2016). STMicroelectronics AN4427 Application Note. Gasket Design for Optimal Acoustic Performance in MEMS Microphones. https://www.st.com/resource/en/application_note/an4427-gasket-design-for-optimal-acoustic-performance-in-mems-microphones-stmicroelectronics.pdf.

[31] Homentcovschi D., Miles R.N. (2011). An analytical-numerical method for determining the mechanical response of a condenser microphone. J. Acoust. Soc. Am..

[32] Zuckerwar A.J. (1978). Theoretical response of condenser microphones. J. Acoust. Soc. Am..

[33] Prato A., Montali N., Guglielmone C., Schiavi A. (2018). Pressure calibration of a digital microelectromechanical system microphone by comparison. J. Acoust. Soc. Am..

